# Audit of High Dose Antipsychotic Therapy (HDAT) Prescribing Among Inpatients in East Suffolk

**DOI:** 10.1192/bjo.2024.530

**Published:** 2024-08-01

**Authors:** Shamim Ruhi, Anne Alase, Motolani Aregbesola, Quratulain Khan, Fariha Rizvi

**Affiliations:** 1 Norfolk and Suffolk NHS Foundation Trust, Ipswich, United Kingdom; 2 Leeds and York Partnership NHS Foundation Trust, Leeds, United Kingdom

## Abstract

**Aims:**

High doses of antipsychotic therapy (HDAT) are often prescribed in secondary mental health services and has been the subject of many audits and service improvements. This interest is largely due to the increased morbidity and mortality related to HDAT, and strong advocacy for clear rationales to guide this decision. There is a need for continuous review and monitoring to prevent unnecessary prescribing.

Our audit was used to determine the prevalence of HDAT in East Suffolk inpatient settings and assess whether review planning and monitoring of HDAT was practiced.

Standards for antipsychotic dosage were established using British National Formulary and Maudsley Prescribing Guidelines for Psychiatry.

**Methods:**

Retrospective data was collected using electronic records of patients 18 years and above who were discharged from inpatient psychiatric wards located in East Suffolk between 1st July and 31st December 2021.

Data available included discharge medication letters, discharge summaries and inpatient clinical notes.

**Results:**

A total of 256 patients were discharged from East Suffolk wards in the 6-month period between 1st July and 31st December 2021.

Majority of the patients (80%) were above 65 years of age with more than half of patients being male 114 (56.3%).

Ninety-seven (37.9%) patients had a diagnosis of schizophrenia or schizophrenia-like and delusional disorders, while approximately 25% of the audited population had a mood disorder.

9% had a singular diagnosis of personality disorder.

One hundred and sixty-six (64.6%) patients were on antipsychotic medications and two (1.2%) patients were discharged on HDAT.

**Conclusion:**

High dose antipsychotic prescribing was not as prevalent as initially assumed. This audit noted only one of the two patients on HDAT did not have the appropriate monitoring form completed.

Good clinical practice recommends the need for the completion of a high dose antipsychotic therapy (HDAT) form for each patient, which would allow proper monitoring.

